# Alexis® Orthopedic Protector Provides Wound Protection and Aids in Hypertrophic Scar Prevention in Total Hip Arthroplasty

**DOI:** 10.7759/cureus.63545

**Published:** 2024-06-30

**Authors:** Shuro Furuichi, Shigeru Mitani, Toyohiro Kawamoto, Ryosuke Kikuoka, Yuki Ota

**Affiliations:** 1 Department of Bone and Joint Surgery, Kawasaki Medical School, Okayama, JPN

**Keywords:** operation scar, keloid, skin protection, anterolateral supine approach, wound protector, total hip arthroplasty

## Abstract

Purpose: Total hip arthroplasty (THA) is one of the most widely performed orthopedic surgeries. Techniques for small skin incisions and preservation of muscles and tendons have been developed. However, avoiding skin complications and muscle damage due to forced deployment and surgical manipulation is challenging. This study aimed to investigate whether the use of Alexis^®^ Orthopedic Protector (Applied Medical Resources Corp., Rancho Santa Margarita, CA, USA) affects postoperative outcomes.

Methods: This was a retrospective cohort study including 118 patients who underwent primary THA by the same surgeon at our single institution between March 2021 and March 2023. Protectors were used alternately for each operation. Fifty-nine patients were in the protector-using group (P group), and 59 were in the nonprotector-using group (N group), with comparisons made between groups. Protectors were placed under the fascia in all patients.

Results: Preoperative blood tests showed no difference in renal and hepatic function between the two groups. No differences in postoperative C-reactive protein (CRP) and creatine kinase (CK) values or in the Japanese Orthopedic Association Hip Disease Evaluation Questionnaire (JHEQ) and Numerical Rating Scale (NRS) scores were observed. Postoperative redness was significantly higher in the N group than in the P group (49.2% vs. 7%). The percentage of hypertrophic scars at three months postoperatively was 18.6% in the N group and 7% in the P group. Furthermore, the Japan Scar Workshop Scar Scale (JSS) indicated that hypertrophic scars were significantly worse in the N group than in the P group (*p* = 0.0012).

Conclusion: Alexis® Orthopedic Protectors can not only provide short-term wound protection but also reduce the rate and degree of hypertrophic scarring.

## Introduction

Minimally invasive surgery has been increasingly used in total hip arthroplasty (THA), and good results have been reported [1]. However, there is a need for smaller skin incisions. Smaller skin incisions and the minimally invasive nature of THA allow for muscle-tendon preservation, and the anterolateral approach is increasingly used as an alternative to the posterior approach. In our clinic, the anterolateral approach is performed on the operated side of the hip joint with a 10 cm skin incision that does not cut the muscles, mainly in the short external rotator muscle, in cases with no previous surgery. However, avoiding skin complications and muscle damage, particularly skin and gluteus medius muscle injuries, caused by forced deployment and surgical manipulation, is challenging.

Small skin incisions may result in delayed wound healing due to skin stress. It has been reported that using the anterolateral approach of intermuscular entry can cause femoral fractures [2], femoral nerve palsy [3], vascular injuries, and injuries to the midline muscle [4] due to the use of retractors. Furthermore, injuries caused by retractors to secure the surgical field, reaming on the acetabular side, or broaching on the femoral side may result in delayed wound healing and a high risk of infection if the wound is severely damaged. Thus, wound protectors have been developed to avoid these problems.

Alexis® Orthopedic Protector (Applied Medical Resources Corp., Rancho Santa Margarita, CA, USA) is considered effective in protecting wounds. The protector is characterized by its wound protection and retraction effect when deploying the acetabular and femoral sides, regardless of the approach. Additionally, the device is made from polyurethane polyester, which is strong, X-ray permeable, and easy to use. Many studies have reported the effectiveness of protectors in reducing surgical site infection (SSI) [5-8], not in protecting wounds. Additionally, only one study has reported the usefulness of protectors in preventing infection in the shoulder joint, but no studies have investigated the use of protectors in the field of orthopedic surgery [9]. Therefore, this study aimed to investigate whether the use of protectors affects the postoperative skin condition, blood test results, and surgery results.

## Materials and methods

Ethical statement

This study was approved by the Institutional Review Board of our institution before implementing the study methods (Approval No. 6132-00) and was conducted according to the World Medical Association Declaration of Helsinki. Patients were given the option to discontinue the study through our website. Since this study is noninvasive and backward-looking, verbal informed consent was obtained under the direction of the Ethics Committee.

Study design and study group

**Figure 1:**
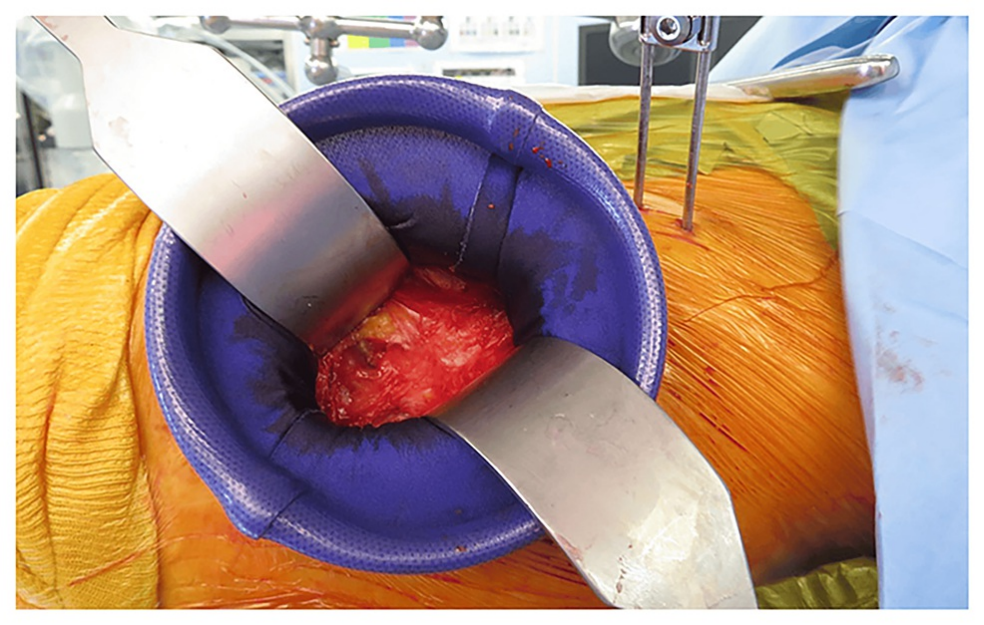
Use of Alexis® Orthopedic Protector. Source: Original work.

This was a retrospective cohort study. A total of 118 patients who underwent their first THA by the same surgeon at our institution between March 2021 and March 2023 were included in this study. Of the 118 patients, 59 were in the protector group (group P) (Figure 1), and 59 were in the non-protector group (group N). A comparison between the groups was performed. Data were extracted from the medical records collected at the time of admission for THA and postoperatively. The exclusion criteria included long skin incisions and invasive surgeries. However, no cases of high dislocation were found during the study period.

Survey and measurement methods

Preoperative patient data, including age at surgery, sex, height, weight, and body mass index (BMI), were collected. The underlying and primary diseases that led to surgery were defined. The levels of albumin, alanine transaminase, aspartate transaminase, γ-glutamyl transpeptidase, estimated glomerular filtration rate, and creatinine were investigated from the preoperative blood tests. The surgical approach, prosthesis model, operative time, blood loss, and length of hospital stay were investigated. Clinical findings were assessed two weeks after surgery using the Japanese Orthopedic Association Hip Disease Evaluation Questionnaire (JHEQ) [10], a patient-based evaluation tool, and the Numerical Rating Scale (NRS), a pain assessment tool. C-reactive protein (CRP) (mg/dL) and creatine kinase (CK) (U/L) were measured as postoperative blood tests. Postoperative measurements were performed on the day after surgery and after two weeks.

**Figure 2:**
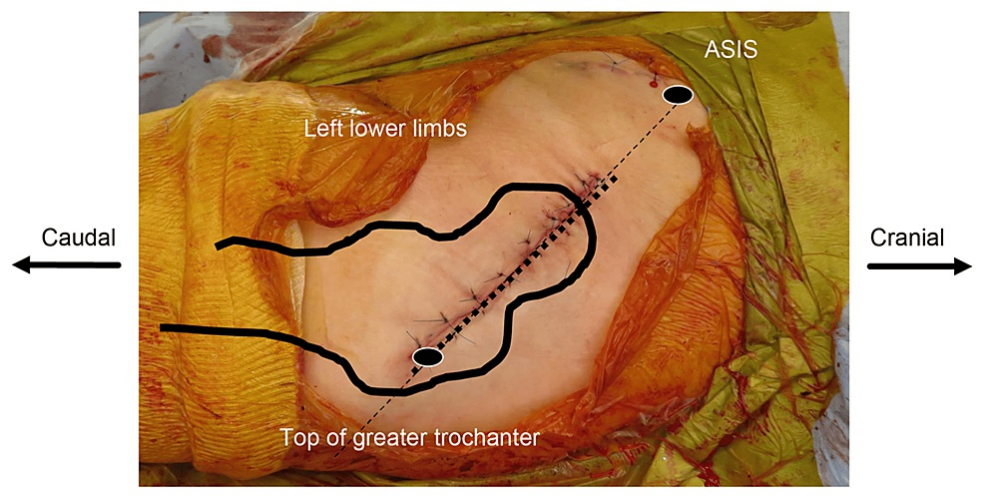
Position of the skin incision. Source: Original work.

Photographs of the skin condition were taken, and the skin condition was examined. The surgical incision was made using an anterolateral approach in the supine position. A posterior approach was used for patients with a high possibility of dissection from the normal anatomy (i.e., those with a history of surgery on the operative hip joint) because it was easier to obtain deployment. For the anterolateral approach, the skin incision was 10 cm on a line passing from the anterior superior iliac spine to the top of the greater trochanter, whereas for the posterior approach, the skin incision was 10 cm from the top of the greater trochanter to the posterior superior iliac spine (Figure 2). In obese patients, an additional incision of a few centimeters was made at the surgeon's discretion.

**Figure 3:**
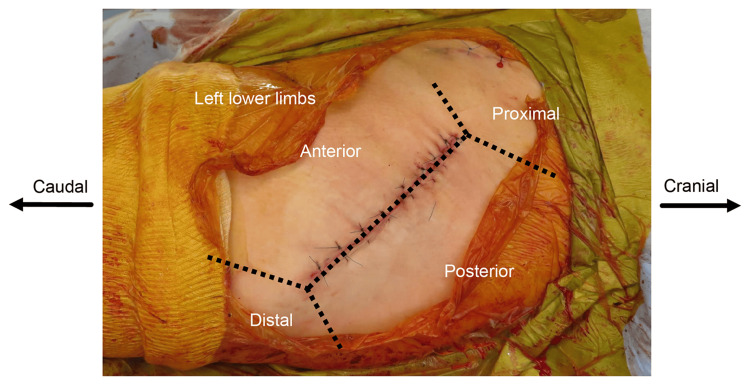
Position of redness. Source: Original work.

The presence of redness was investigated immediately after surgery. The location of the wound was checked by checking the condition of the 10 cm skin margin, which was divided into proximal, distal, anterior, and posterior (Figure 3), to examine whether redness was present in any of the areas. Skin fusion was checked for white necrosis two weeks after surgery. Whether hypertrophic scars had developed was investigated three months after surgery. Evaluations were performed visually, and judgment was made if at least four out of five orthopedic surgeons agreed that it was present. Hypertrophic scars were evaluated using the Japan Scar Workshop Scar Scale (JSS) [11], which consists of two parts: risk factors and present symptoms, including 12 items. The total score is 25 points, with 0-5 points indicating low risk (matured scars), 6-15 points indicating intermediate risk (hypertrophic scars), and 16-25 points indicating high risk (keloids). The occurrence of postoperative SSI was also investigated.

The implants used were tapered wedge short stems (Initia®/J-Taper®, Kyocera, Kyoto, Japan; Taperloc Microplasty®, Zimmer Biomet, Warsaw, IN, USA). For patients with poor bone quality, Full HA stem (Avenir®, Zimmer Biomet, Warsaw, IN, USA/Universia®, Teijin Nakashima Medical, Okayama, Japan) or Zweymüller-type stem (Inheritor®, Kyocera, Kyoto, Japan) were selected. For patients with an anteversion angle greater than 60°, a modular-type stem (S-ROM®, DePuy Synthes, Warsaw, IN, USA) with an adjustable anteversion angle was used.

**Figure 4:**
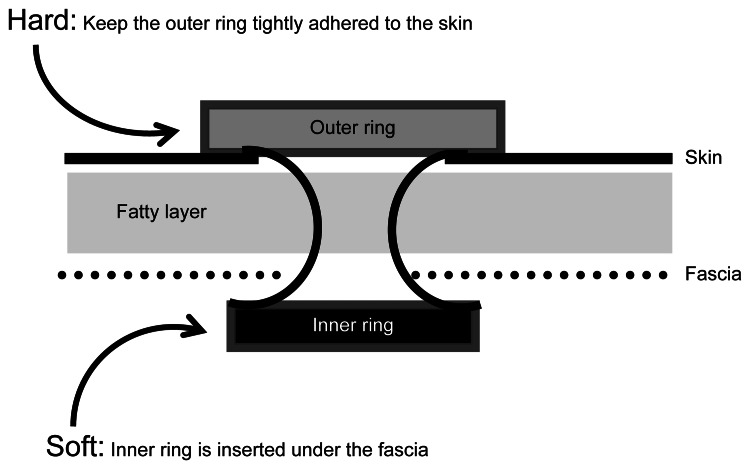
Application of Alexis® Orthopedic Protector. Source: Original work. Image Credits: Shuro Furuichi.

Iodine-impregnated drapes were used for wound protection and infection prevention. Patients were under general anesthesia and placed in a cleansing position. Patients were positioned as necessary for the operation. The nurse, who was not clean, lifted one leg and disinfected it with iodine. Only one side was performed, from below the navel to the toes. An iodine drape was applied directly above the surgical wound to keep the area clean (Figure 4).

Protector removal was performed just before wound closure. In all cases, the fascia and subcutaneous fat were sutured with No. 1 Vicryl® (Ethicon, Johnson & Johnson, New Brunswick, NJ), and the subcutaneous tissue was sutured with No. 3-0 Vicryl® (Ethicon, Johnson & Johnson). Simple sutures were made with 3-0 nylon thread at a pitch of 1 cm. All patients were cleaned and disinfected after surgery with 0.05% chlorhexidine gluconate. Finally, the procedure was terminated by covering the wound with Leukomed Control® (Terumo, Tokyo, Japan), a wound dressing large enough to completely cover the wound. The wound was evaluated two days, two weeks, and three months after surgery. Stitches were removed two weeks after surgery, the wound was covered with a film agent, and the covering material was removed three weeks after surgery in all cases. The patient was discharged home two weeks after surgery.

Perioperative antimicrobial prophylaxis with cefazolin sodium (1 g) was administered to all patients 30 minutes before the start of the surgical procedure. After surgery, another antimicrobial infusion was administered on the day of surgery, and antimicrobial therapy was completed with three doses of cefazolin sodium (1 g) three times per day on the following day. Analgesics with celecoxib (100 mg) were uniformly administered two times per day, starting the day after surgery.

Statistical analysis

Continuous and categorical variables were presented as mean ± standard deviation, frequency, and percentage. Continuous variables were compared between the two groups using the Mann-Whitney U-test. Categorical variables were tested using the chi-square test. All statistical analyses were performed using Stat Flex Version 7 (Artech Co. Ltd., Osaka, Japan). A p-value of <0.05 indicated a significant difference.

## Results

No significant differences in age, sex, height, weight, or BMI were observed between the two groups at the time of surgery. The primary and underlying diseases that led to surgery were similar in both groups. Osteoarthritis was more common in both groups. Furthermore, there was no disease specificity (Table 1). Preoperative blood tests revealed that the levels of albumin, alanine transaminase, aspartate transaminase, γ-glutamyl transpeptidase, estimated glomerular filtration rate, and creatinine were not different between the two groups (Table 2). Additionally, the operative time, blood loss, and hospital stay were not different between the two groups. A short stem was the most common surgical approach, and joint prostheses were used, accounting for more than 80% of the cases (Table 3). The JHEQ score was 47.9 ± 12.6 in the N group and 48.3 ± 11.9 in the P group two weeks after surgery, with no difference between the two groups. The NRS score was 1.3 ± 1.4 in the N group and 0.9 ± 1.1 in the P group, with no difference between the two groups. No statistical differences in CRP and CK were observed between the day after surgery and 2 weeks after surgery (Table 4).

**Table 1:** Characteristics of patients in the N and P groups. *Mann–Whitney U-test. **Chi-square test. BMI: Body mass index. Blank columns in the table are hyphenated because the number of cases was small and significance tests were not conducted.

Patient characteristics	Group N (n = 59)	Group P (n = 59)	p-value
Age at the time of surgery	63.8 ± 10.9	65.6 ± 10.5	0.23177^*^
Male-to-female ratio	8:51	13:46	0.22882^**^
Height	156.4 ± 8.3	157.8 ± 8.8	0.34617^*^
Weight	60.7 ± 12.8	61.7 ± 9.7	0.53945^*^
BMI	24.4 ± 4.6	24.8 ± 3.7	0.92282^*^
Primary disease	OA (n = 50)	OA (n = 48)	-
	ION (n = 9)	ION (n = 9)	
	-	RA (n = 2)	
Underlying disease	Rheumatoid arthritis (n = 2)	Rheumatoid arthritis (n = 2)	-
	Cerebral palsy (n = 1) Diabetes mellitus (n = 1)	Diabetes mellitus (n = 2)	-
	Heart disease (n = 1)	Alcoholic liver disease (n = 1)	-
	Alcoholic liver disease (n = 1)		-

**Table 2:** Blood tests for patients in the N and P groups. *Mann–Whitney U-test.

Test items	Standard value	Group N (n = 59)	Group P (n = 59)	p-value
Albumin (g/dL)	4.1–5.1	4.1 ± 0.4	4.1 ± 0.4	0.50768^*^
Alanine transaminase (U/L)	10–42	18.1 ± 14.7	20.6 ± 12.3	0.56792^*^
Aspartate transaminase (U/L)	13–30	20.9 ± 7.2	24.1 ± 15	0.49318^*^
γ-Glutamyl transpeptidase (U/L)	13–64	42.6 ± 67.2	55.4 ± 147.2	0.13630^*^
Estimated glomerular filtration rate (mL/min/1.73 m^2^)	>60	70.8 ± 16.5	69.5 ± 16.5	0.75695^*^
Creatinine (mg/dL)	0.65–1.07	0.7 ± 0.2	0.8 ± 0.3	0.82946^*^

**Table 3:** Details of the surgery and hospital stay. *Mann–Whitney U-test. AL: anterolateral approach; PL: posterolateral approach. Blank columns in the table are hyphenated because the number of cases was small and significance tests were not conducted.

Intraoperative and postoperative	Group N (n = 59)	Group P (n = 59)	p-value
Operation time (min)	81.1 ± 18	79.2 ± 18.2	0.85686^*^
Blood loss (g)	318.3 ± 139	308.5 ± 168	0.38895^*^
Hospital stay (days)	20.4 ± 7.7	19.7 ± 7.3	0.46519^**^
Intraoperative approach	AL (56 (95%)) PL (3 (5%))	AL (57 (97%)) PL (2 (3%))	-
Models used	Short (49 (83%))	Short (46 (78%))	-
	Full HA (8 (14%))	Full HA (10 (17%))	
	Zweymüller (1 (1.5%))	Zweymüller (2 (3.4%))	-
	Modular (1 (1.5%))	Modular (1 (1.5%))	-

**Table 4:** Postoperative clinical evaluation and blood tests. *Mann–Whitney U-test. JHEQ: Japanese Orthopaedic Association Hip Disease Evaluation Questionnaire; NRS: Numerical Rating Scale; CRP: C-reactive protein; CK: creatine kinase.

Postoperative pain and laboratory values	Group N (n = 59)	Group P (n = 59)	p-value
JHEQ 2 weeks postoperatively	47.9 ± 12.6	48.3 ± 11.9	0.80217^*^
NRS 2 weeks postoperatively	1.3 ± 1.4	0.9 ± 1.1	0.26551^*^
CRP on the day after surgery	5.3 ± 2.5	5.1 ± 2.7	0.55923^*^
CRP 2 weeks postoperatively	0.7 ± 0.7	0.8 ± 1.2	0.33273^*^
CK on the day after surgery	592.7 ± 286	574.7 ± 278	0.73657^*^
CK 2 weeks postoperatively	76 ± 70.2	83.8 ± 96.7	0.81293^*^

Immediate postoperative redness was observed in 29 (49.2%) and 10 (17%) patients in the N and P groups, respectively, which was significantly greater in the N group (p = 0.00020). Considering the obese cases with a BMI of 30 or more, two out of eight cases in the N group and one out of six cases in the P group, the skin redness was not strong in the obese cases. Furthermore, there were no significant differences in the percentage of patients with white necrosis two weeks after surgery; however, the proportion of patients with hypertrophic scars at three months postoperatively was significantly lower in the P group than in the N group.

However, the JSS showed that hypertrophic scars were significantly more severe in the N group than in the P group (p = 0.00063) (Table 5). The difference was even more pronounced in cases with hypertrophic scars. In the N group, 11 patients had a JSS score of 2.8 ± 2.4, and in the P group, 10 patients had a JSS score of 1.8 ± 1.3. In the P group, nine patients had mild scars, and one patient had a moderate scar, whereas in the N group, the severity was worse.

**Table 5:** Postoperative wound condition. *Mann–Whitney U-test; **chi-squared test. JSS: Japan Scar Workshop Scar Scale.

Wound condition	Group N (n = 59)	Group P (n = 59)	p-value
Redness immediately after surgery	29 (49.2%)	4 (7%)	0.00020^**^
White necrosis 2 weeks after surgery	8 (14%)	5 (8%)	0.37775^**^
Hypertrophic scar 3 months after surgery	11 (18.6%)	4 (7%)	0.05^**^
Hypertrophic scar JSS score	2.8 ± 2.4	1.8 ± 1.3	0.00063^*^

Of the 29 patients with redness in the N group, 3 (10.3%) had hypertrophic scars, whereas all 4 (100%) patients in the P group had hypertrophic scars. The JSS score of the patients with hypertrophic scars was 7.3 ± 1.3 in the N group and 4.5 ± 0.8 in the P group, with the N group being significantly worse (p = 0.00012) (Table 6). In both groups, redness was more common on the proximal side (Table 7). No cases of infection, wound dehiscence, delayed wound healing, complications, or protector breakage were observed.

**Table 6:** JSS in cases of hypertrophic scarring. *Mann–Whitney U-test JSS: Japan Scar Workshop Scar Scale.

JSS Score	Group N (n = 11)	Group P (n = 4)	p-value
Hypertrophic scar JSS score	7.3 ± 1.3	4.5 ± 0.8	0.00012*

**Table 7:** Location and frequency of redness immediately after surgery.

Position	Group N (n = 29)	Group P (n = 4)
Proximal	17 (59%)	4 (100%)
Distal	5 (17%)	0
Proximal + distal	0	0
Proximal + anterior	1 (3%)	0
Proximal + posterior	3 (11%)	0
Distal + anterior	1 (3%)	0
Anterior	0	0
Posterior	2 (7%)	0
Anterior + posterior	0	0

Case 1: A 66-year-old female (N group)

**Figure 5:**
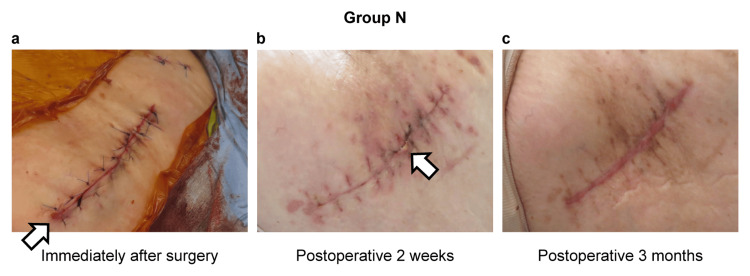
A 66-year-old female, group N. Source: Original work. (a) Redness was observed distally immediately after surgery; (b) white necrosis was observed posteriorly two weeks after surgery; (c) moderate scarring was observed three months after surgery.

A 66-year-old female patient with left hip osteoarthritis underwent left THA. The patient had no underlying disease. Her height was 148.5 cm, her weight was 59.9 kg, and her BMI was 27.2 kg/m^2^. All preoperative blood tests were normal. An anterolateral approach was performed using a short stem without a protector. Redness was observed distally immediately after surgery. The operation time was 67 minutes, and the blood loss was 353 g. The patient was hospitalized for 16 days. The NRS and JHEQ scores were 0 and 41, respectively, two weeks after surgery. CRP was 3.7 (mg/dL), and CK was 348 (U/L) the day after surgery. A white necrotic area was observed after suture removal two weeks after surgery. The patient was clinically ambulatory three months after surgery but reported discomfort in the wound. The JSS score was 10 points, indicating moderate scarring (Figure 5).

Case 2: A 48-year-old female (P group)

**Figure 6:**
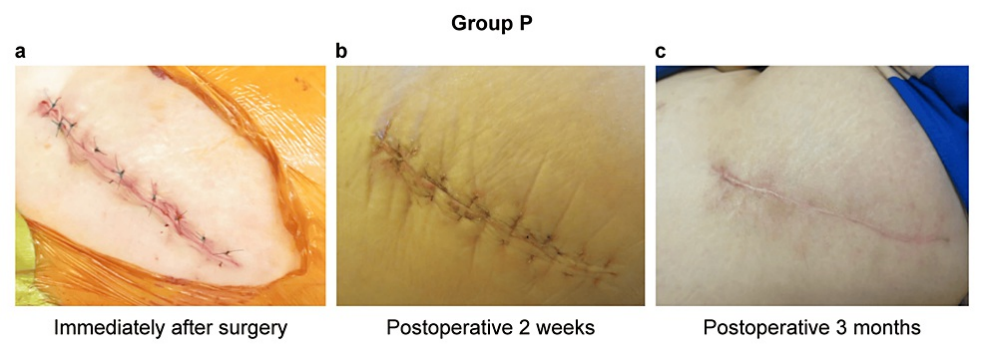
A 48-year-old woman, group P. Source: Original work. (a) No redness was observed immediately after surgery; (b) no white necrosis was observed two weeks after surgery; (c) no hypertrophic scar was observed three months after surgery.

A 48-year-old female patient with right hip osteoarthritis underwent right THA. Her height was 156.5 cm, her weight was 68.8 kg, and her BMI was 28.1 kg/m^2^. All preoperative blood tests were normal. An anterolateral approach was performed using a short stem with a protector. No redness in the immediate postoperative period was observed. The operative time was 90 minutes, and the blood loss was 150 g. The patient was hospitalized for 16 days. The NRS and JHEQ scores were 1 and 62, respectively, two weeks after surgery. CRP and CK were 3.61 mg/dL and 651 U/L, respectively, on the day after surgery, and CRP and CK were 0.56 mg/dL and 63 U/L, respectively, two weeks after surgery. No white necrosis was observed after suture removal two weeks after surgery, and no clinical symptoms were observed three months after surgery. The JSS score was 2 points, indicating mild scarring (Figure 6).

## Discussion

In this study, no difference in age or body weight was observed between the P group that used protectors and the N group that did not. Additionally, primary and underlying diseases were similar between the two groups. The preoperative blood test values did not change. Therefore, no bias in patient characteristics was observed. Similarly, no differences in the operative time or blood loss were observed. The wearing time of the protector used was approximately 30 seconds in all cases, and the use of the protector did not prolong the operative time. The protector was applied under tension, which was thought to reduce bleeding in the subcutaneous and fatty layers. However, the amount of bleeding did not differ between the two groups. This may be because we used an electrocautery scalpel to carefully stop bleeding when expanding the subcutaneous and fatty layers.

All patients were scheduled for suture removal two weeks after surgery, and both groups were discharged home around 20 days after suture removal. No difference in the timing of discharge was observed between the two groups. The NRS and JHEQ scores did not differ between the two groups. Furthermore, we considered that wound protection may be associated with function and pain. However, no difference was observed two weeks after surgery. CRP reflects inflammatory findings, including wound and skin inflammation, and CK reflects muscle mass. We thought that skin and muscle damage would differ with the use of protectors. However, blood tests showed no differences, and the same was true at two weeks.

In the anterolateral approach, the retractor provides muscle protection to the vastus medialis and tensor fascia femoris. However, the protector alone may not completely preserve the muscle tissue itself. Reports of skin damage due to THA have shown that minimally invasive THA causes many skin complications and scarring [12,13]. These skin problems may increase due to forced stretching of the skin. In this study, postoperative redness was significantly greater in the N group. The protector itself protects the wound. The site of the redness was more proximal, but there was no other tendency. Proximal redness is thought to be the site where the femoral retractor or broach strikes. We suggest that redness can be prevented by using this protector.

**Figure 7:**
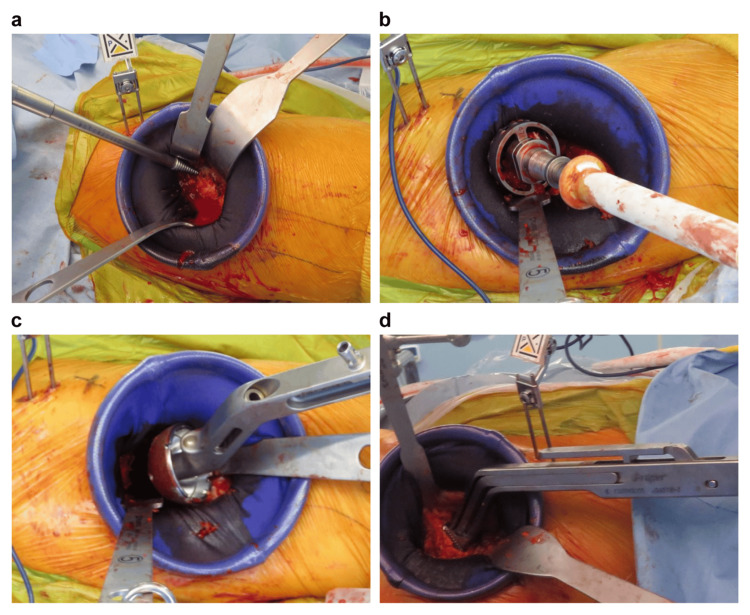
Intraoperative operation with a high risk of skin damage. Source: Original work. (a) Removal of the head using a corkscrew; (b) acetabular reaming; (c) insertion of the cup into the acetabulum; (d) stem insertion into the femur.

The skin condition may change depending on the approach and the model used. Regarding the approach, the skin condition of the three patients in the N group and the two patients in the P group who used the posterior approach showed only one case of redness in the P group and one case of hypertrophic scar in the N group. Regarding the model, the skin condition of the 10 patients in the P group and the 13 patients in the N group who used Full HA, Zweymüller, and modular stems showed that redness occurred in three patients in the P group and one patient in the N group, white necrosis occurred in one patient in the P group, and one patient in the N group, and thickened scars occurred in two patients in the P group and two patients in the N group. The most common use of corkscrews is for extraction of the head, reaming, rasping, and other repetitive operations, as well as for cup placement. Skin contamination can be avoided without stress during cup placement, which requires cleanliness procedures (Figure 7). This is particularly useful in each step of the THA.

In this study, the use of protectors reduced redness immediately after surgery and reduced the degree of hypertrophic scarring. However, the percentage of hypertrophic scars at three months postoperatively was 18.6% in the no-use group and 7% in the under-use group. The cause of these hypertrophic scars is unknown, but cytokines, such as TGF-β1 and IGF-1, have been implicated [14,15]. Therefore, we expected that there would be a difference in the inflammatory response. However, no difference was observed in this study. These findings are similar to those of a previous report that investigated CRP with a minimally invasive approach [16]. The inflammatory response decreased and improved in almost all patients approximately two weeks after surgery, regardless of keloid formation.

Obese patients may have experienced excessive pressure on their skin due to a uniform 10 cm skin incision. Considering the cases with a BMI over 30, two out of eight cases in the N group and one out of six cases in the P group, it is unlikely that the tendency to obesity affected skin redness. However, while only 10% of the patients in the N group developed redness, all patients in the P group developed hypertrophic scars. This may be due to the excessive load of the retractor and the open wound device used for artificial joints in cases where redness could be seen despite the use of protectors. In these cases, the skin should have been cut as large as possible without regard to the incision size. The most important finding of this study is that the use of protectors can reduce the frequency and extent of hypertrophic scarring. Such a reduction in the extent of hypertrophic scarring has not been previously reported. Furthermore, protectors can reduce the degree of erythema and, consequently, the degree of hypertrophic scarring. Skin incision is one of the parameters for successful hip arthroplasty. Obese patients have more subcutaneous fat, which may make surgery more difficult. However, it is important not to be overly concerned with the length of the skin incision, especially in obese patients.

In all cases, the overall rate of skin redness was low. Thus, the use of protectors can lead to a reduction in skin problems immediately after surgery. The use of protectors eliminates the need for frequent wound margin checks by the medical staff and is expected to improve patient satisfaction. Previous studies have reported that the incidence of surface and deep SSI in initial THA was 0.2% and 3.4%, respectively [17,18]. In this study, no postoperative infection was observed. One of the reasons for this may be the small number of cases. Overseas reports on protectors and infection have shown that protectors significantly reduce SSI in the surgical field [5,6]. Similarly, studies in Japan have reported good results, showing that protectors reduce SSI due to indigenous bacteria in the surgical field [7]. However, delayed wound healing may trigger infection. Fukuda et al. reported that the occurrence of postoperative infections can prolong hospitalization and require significant medical resources [19]. Thus, efforts should be made to reduce postoperative infection using protectors because prolonged hospitalization can significantly decrease patient satisfaction. The difference between these reports in gastrointestinal surgery and orthopedic surgery is the risk of infection due to the presence of commensal bacteria.

This study has some limitations. First, the number of patients was small, and determining whether the protectors were effective in reducing infection was not possible. Additionally, this was not a randomized controlled study but an alternation of consecutive surgeries. Second, not all patients in this study used the same approach. Some patients used the posterior approach, and some used a stem other than a tapered wedge or short stem. Hypertrophic scars are well-defined, localized nodules in the wound area. However, evaluation of subsequent exacerbations or remissions was not possible due to the relatively short observation period (three months postoperatively).

Despite these limitations, this study showed that the protector provided intraoperative wound protection, reduced redness, and prevented infection. It was possible to reduce the percentage of hypertrophic scars in the short term, and the degree of hypertrophic scarring was also reduced using protectors.

## Conclusions

Alexis® Orthopedic Protector is expected to provide wound protection. Comparisons of postoperative results between patients with and without protectors showed that the percentage of hypertrophic scars was lower in the P group than in the N group, and JSS was less severe with the use of protectors. Regardless of the approach, no protector suffered damage. The use of protectors can not only provide short-term wound protection but also reduce the rate and degree of hypertrophic scarring.

## References

[1] DiGioia AM 3rd, Plakseychuk AY, Levison TJ, Jaramaz B (2003). Mini-incision technique for total hip arthroplasty with navigation. J Arthroplasty.

[2] Miettinen SS, Mäkinen TJ, Kostensalo I, Mäkelä K, Huhtala H, Kettunen JS, Remes V (2016). Risk factors for intraoperative calcar fracture in cementless total hip arthroplasty. Acta Orthop.

[3] Takada R, Jinno T, Miyatake K (2018). Direct anterior versus anterolateral approach in one-stage supine total hip arthroplasty. Focused on nerve injury: a prospective, randomized, controlled trial. J Orthop Sci.

[4] van Oldenrijk J, Hoogland PV, Tuijthof GJ, Corveleijn R, Noordenbos TW, Schafroth MU (2010). Soft tissue damage after minimally invasive THA. Acta Orthop.

[5] Reid K, Pockney P, Draganic B, Smith SR (2010). Barrier wound protection decreases surgical site infection in open elective colorectal surgery: a randomized clinical trial. Dis Colon Rectum.

[6] Lee P (2009). Use of wound-protection system and postoperative wound-infection rates in open appendectomy. Arch Surg.

[7] Horiuchi T, Tanishima H, Tamagawa K (2007). Randomized, controlled investigation of the anti-infective properties of the Alexis retractor/protector of incision sites. J Trauma.

[8] Cheng KP, Roslani AC, Sehha N, Kueh JH, Law CW, Chong HY, Arumugam K (2012). ALEXIS O-Ring wound retractor vs conventional wound protection for the prevention of surgical site infections in colorectal resections(1). Colorectal Dis.

[9] Smith ML, Gotmaker R, Hoy GA, Ek ET, Carr A, Flynn JN, Evans MC (2018). Minimizing Propionibacterium acnes contamination in shoulder arthroplasty: use of a wound protector. ANZ J Surg.

[10] Matsumoto T, Kaneuji A, Hiejima Y (2012). Japanese Orthopaedic Association Hip Disease Evaluation Questionnaire (JHEQ): a patient-based evaluation tool for hip-joint disease. The Subcommittee on Hip Disease Evaluation of the Clinical Outcome Committee of the Japanese Orthopaedic Association. J Orthop Sci.

[11] Ogawa R, Akita S, Akaishi S (2019). Diagnosis and treatment of keloids and hypertrophic scars-Japan scar workshop consensus document 2018. Burns Trauma.

[12] Woolson ST, Mow CS, Syquia JF, Lannin JV, Schurman DJ (2004). Comparison of primary total hip replacements performed with a standard incision or a mini-incision. J Bone Joint Surg Am.

[13] Goldstein WM, Branson JJ, Berland KA, Gordon AC (2003). Minimal-incision total hip arthroplasty. J Bone Joint Surg Am.

[14] Peltonen J, Hsiao LL, Jaakkola S (1991). Activation of collagen gene expression in keloids: co-localization of type I and VI collagen and transforming growth factor-beta 1 mRNA. J Invest Dermatol.

[15] Daian T, Ohtsuru A, Rogounovitch T (2003). Insulin-like growth factor-I enhances transforming growth factor-beta-induced extracellular matrix protein production through the P38/activating transcription factor-2 signaling pathway in keloid fibroblasts. J Invest Dermatol.

[16] Tottas S, Tsigalou C, Ververidis A (2020). Supercapsular percutaneously assisted total hip arthroplasty versus lateral approach in total hip replacement. A prospective comparative study. J Orthop.

[17] Phillips CB, Barrett JA, Losina E (2003). Incidence rates of dislocation, pulmonary embolism, and deep infection during the first six months after elective total hip replacement. J Bone Joint Surg Am.

[18] Poon PC, Rennie J, Gray DH (2001). Review of total hip replacement. The Middlemore Hospital experience, 1980-1991. N Z Med J.

[19] Fukuda H, Sato D, Iwamoto T, Yamada K, Matsushita K (2020). Healthcare resources attributable to methicillin-resistant Staphylococcus aureus orthopedic surgical site infections. Sci Rep.

